# Electromyogram-Based Classification of Hand and Finger Gestures Using Artificial Neural Networks

**DOI:** 10.3390/s22010225

**Published:** 2021-12-29

**Authors:** Kyung Hyun Lee, Ji Young Min, Sangwon Byun

**Affiliations:** Department of Electronics Engineering, Incheon National University, Incheon 22012, Korea; lkh256@inu.ac.kr (K.H.L.); wnet50094@gmail.com (J.Y.M.)

**Keywords:** electromyogram, EMG, machine learning, physiological signal, hand-finger movement, gesture recognition, classification, time-domain features, artificial neural network, prosthetic hand

## Abstract

Electromyogram (EMG) signals have been increasingly used for hand and finger gesture recognition. However, most studies have focused on the wrist and whole-hand gestures and not on individual finger (IF) gestures, which are considered more challenging. In this study, we develop EMG-based hand/finger gesture classifiers based on fixed electrode placement using machine learning methods. Ten healthy subjects performed ten hand/finger gestures, including seven IF gestures. EMG signals were measured from three channels, and six time-domain (TD) features were extracted from each channel. A total of 18 features was used to build personalized classifiers for ten gestures with an artificial neural network (ANN), a support vector machine (SVM), a random forest (RF), and a logistic regression (LR). The ANN, SVM, RF, and LR achieved mean accuracies of 0.940, 0.876, 0.831, and 0.539, respectively. One-way analyses of variance and F-tests showed that the ANN achieved the highest mean accuracy and the lowest inter-subject variance in the accuracy, respectively, suggesting that it was the least affected by individual variability in EMG signals. Using only TD features, we achieved a higher ratio of gestures to channels than other similar studies, suggesting that the proposed method can improve the system usability and reduce the computational burden.

## 1. Introduction

For decades, electromyograms (EMGs) have been employed to control prosthetic limbs, such as hands and wrists. In theory, EMG signals recorded from specific muscles associated with hand and finger gestures can be used to control a variety of movements. However, individual finger (IF) gestures are considered to be more difficult to classify than whole-hand and wrist gestures due to the complexity and subtlety of muscle usage for IF movements [1]. Most finger gesture prediction models rely on EMG signals from a large number of channels, which results in the high cost and complexity of the system [2]. For these reasons, many previous studies focused on classifying whole-hand or wrist gestures [3,4,5,6], but recent advances in computing power and machine learning algorithms have allowed the classification of IF gestures with a small number of channels without sacrificing the accuracy or response time.

Feature extraction is an important process for gesture recognition systems to extract the critical information hidden in the raw EMG signals [7]. EMG features are extracted from three domains, namely the time domain (TD), the frequency domain (FD), and the time–frequency domain (TFD) [7], each of which have advantages and disadvantages in gesture classification. Features in the TD are fast and easy to implement because they do not require additional transformation and are calculated directly from the raw EMG signals [7]. In addition, previous studies suggested that the TD features can represent the transient state of the gestures well [8,9,10]. However, the TD features are prone to errors due to the non-stationarity of the EMG signals [11]. Efforts have been made to improve the performance by extracting features from different domains, but this can result in an increase in computational cost.

The response time of the gesture recognition system needs to be short enough to be perceived as real-time recognition by users. Because this latency includes the time required to extract features, simpler implementation of TD features can be beneficial to reduce the total response time, which is one of the strengths of using the TD features in gesture classification. Therefore, TD features have been widely tested for EMG-based hand/finger gesture recognition, along with FD or TFD features [12,13,14,15,16,17,18]. Various machine learning methods, including support vector machine (SVM), k-nearest neighbors (KNN), artificial neural network (ANN), convolutional neural network (CNN), and probabilistic neural network (PNN), have been implemented as classification algorithms. Most studies achieved at least a 90% accuracy in classifying four to ten hand/finger gestures [12,13,14,15,16,17,18]. Although these studies demonstrated successful recognition of gestures, the achievement of both high accuracy and low response time is still a challenge.

Particularly, ANN algorithms have been extensively tested in EMG-based studies, which investigated various types of EMG data for a wide range of applications. For example, some studies tested both TD and FD features as input data [19,20], and others applied raw EMG signals directly to the ANN without a feature extraction process [21,22]. Additionally, the application of research was not limited to hand/finger gesture recognition but included the prediction of force load [23,24] and the detection of neuromuscular disorders [25]. However, studies that applied ANN algorithms to TD features only, excluding other features or data types, for hand/finger gesture recognition, have not been conducted as extensively as other EMG studies, which used both TD and FD features or raw EMG signals for various purposes [26]. Some studies used commercially available wearables, such as Myo armband, to extract TD features from multi-channel signals and build ANN-based classifiers [27,28,29]. However, it is difficult to specify the position of the electrodes relative to muscles with these wearables. Because information on muscles is crucial for optimizing personalized EMG sensors for users with various physiological conditions, the electrode locations need to be estimated precisely.

Therefore, in this study, we developed a real-time hand/finger gesture recognition system based on fixed electrode placement using only TD features. We employed the ANN and three other popular machine learning algorithms, namely SVM, random forest (RF), and logistic regression (LR), as classifiers, and their performances were statistically compared. A total of ten gestures, including seven IF gestures, were classified. We limited the number of channels used for recording EMG signals to reduce the complexity and improve the usability of the recognition system. Hence, three channels, which are relatively few compared to those used in previous studies [12,13,14,15,16,17,18], were used to record EMG signals from three different muscles on a forearm. Six TD features were extracted from each channel, and therefore, a total of 18 TD features were used as input data. Ten healthy subjects were recruited, and for each subject, personalized classifiers were built and tested.

## 2. Materials and Methods

### 2.1. Participants

EMG data were collected from ten healthy male subjects (mean age ± SD, 24.5 ± 1.5). All subjects were right-handed and did not suffer from any neurological condition. Before the experiment, a guide was provided to acquaint the participants with the experimental procedures, and informed consent was obtained from all subjects. This study was approved by the Institutional Review Board of Incheon National University, Incheon, Korea (No. 7007971-201901-002) and performed according to the relevant guidelines.

### 2.2. Equipment and Software

**Figure 1:**
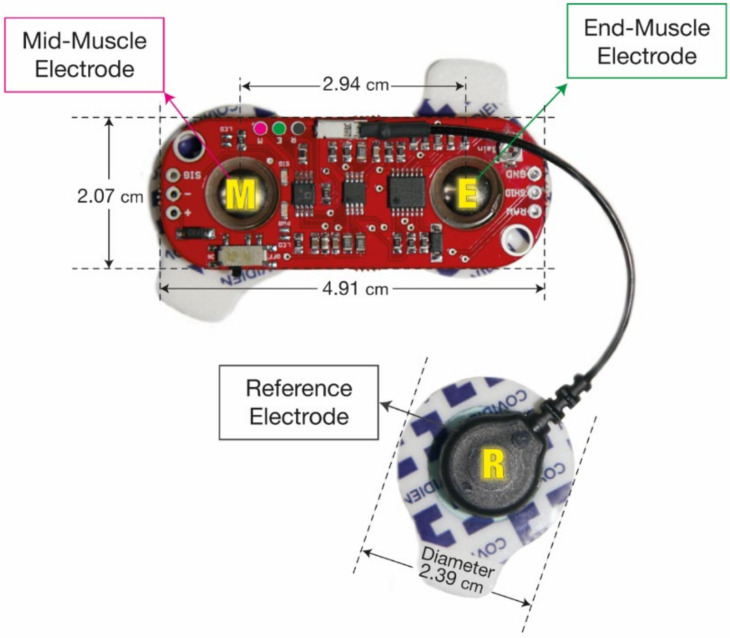
EMG sensor and electrodes used in the experiments. The image is modified from [33].

We followed the guidelines suggested in [30]. All subjects were isolated from the main supply during the experiment. The EMG signals were acquired using MyoWare Muscle sensors (SparkFun Electronics, Niwot, CO, USA) (Figure 1). This sensor has been frequently used in previous EMG studies because of its low cost, easy-to-customize features, and favorable performances reports in validation studies, showing it to be comparable to more expensive commercial EMG systems [31,32]. The sensor is powered by a 5 V supply and consists of three electrodes: mid-muscle, end-muscle, and reference [33]. EMG signals were differentially amplified with adjustable gain of 201*R*_gain_/1 kΩ (CMRR 110 dB, input impedance at 60 Hz is not available). For the electrodes, we used Ag/AgCl electrodes for surface EMG (H124SG, Covidien, Dublin, Ireland), based on conductive and adhesive hydrogel with a 201 mm^2^ gel area, a 251 mm^2^ adhesive area, and an 80 mm^2^ sensor area. Analog EMG signals were collected with a data acquisition (DAQ) system (NI DAQ USB-6361, National Instruments, Austin, TX, USA) to digitize the signals with a sampling rate of 2000 Hz. LabVIEW 2017 (National Instruments) was used to record the signals and remove noise by digital filters. Data processing and machine learning modeling were performed using Python (version 3.7, https://www.python.org/, accessed on 29 December 2021) with scikit-learn (version 0.21.3, https://scikit-learn.org, accessed on 29 December 2021) and TensorFlow (version 2.1.0, https://www.tensorflow.org/, accessed on 29 December 2021). Statistical analyses were performed using R (version 4.0.2, https://www.r-project.org/, accessed on 29 December 2021).

### 2.3. Experimental Setup and Data Acquisition

**Figure 2:**
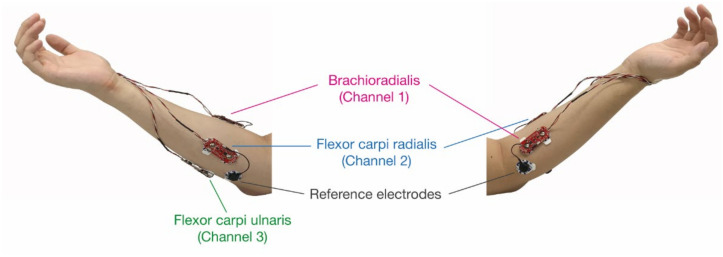
Placement of EMG sensors and electrodes on a forearm.

The EMG sensors were placed on the flexor carpi radialis, flexor carpi ulnaris, and brachioradialis, which are the forearm muscles associated with the selected hand and finger gestures (Figure 2) [1,34]. To improve the EMG signal measurement accuracy, the electrodes were placed on the midline of the muscle belly between an innervation zone and a myotendon junction. The electrodes were placed parallel to the muscle fibers [35]. To determine the attachment location, we used anatomical information and methods recommended in previous literature [36]. After the attachment, electrode placements were confirmed by muscle contraction performed by a subject. Each electrode pair had its reference electrode, which was placed as close to the elbow and as distant from the targeted muscles as possible. The subjects sat in a comfortable chair and relaxed their arm and hand before performing gestures. The entire data acquisition process proceeded for each subject without repositioning the electrodes until the end.

**Figure 3:**
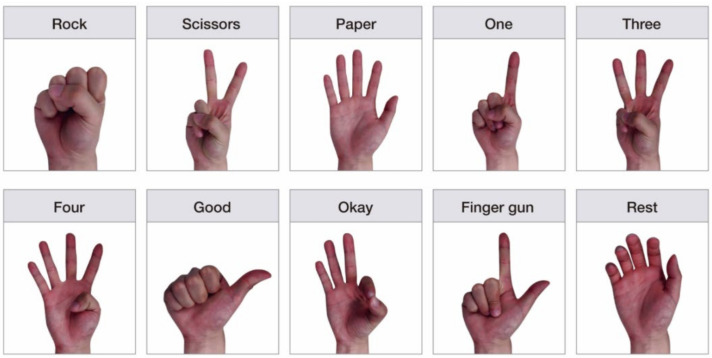
Ten hand and finger gestures used for classification. Two whole-hand gestures (rock and paper) and seven IF gestures (scissors, one, three, four, good, okay, and finger gun) are included.

Ten different gestures—nine non-rest gestures and a rest gesture—were tested and classified as follows: rock, scissors, paper, one, three, four, good, okay, finger gun, and rest (Figure 3). Among the non-rest gestures, rock and paper were considered as whole-hand gestures, and the other seven were considered as IF gestures. A whole-hand gesture is defined as moving all five fingers in the same direction, and the individual finger gesture involves moving at least one finger in a different direction. Although these definitions of finger gestures are not commonly used in the literature, we separated the whole-hand and individual finger gestures to indicate the more complex nature of movements for certain finger gestures. The rock and paper gestures have been regularly included and studied as a finger gesture in previous studies. Therefore, we included rock and paper as common gestures to be compared with other studies.

**Figure 4:**
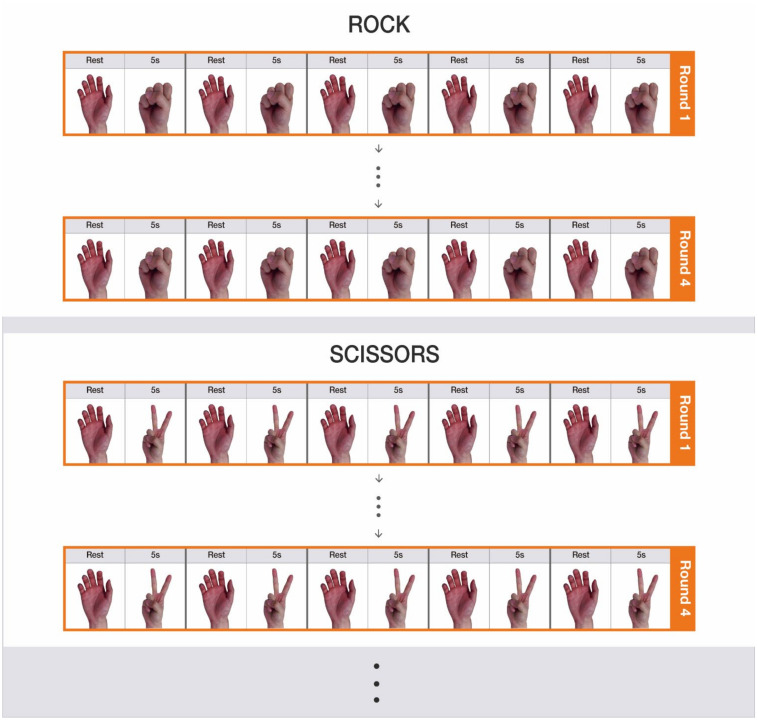
Experimental procedure. Four rounds were conducted for each nine non-rest gesture: rock, scissors, paper, one, three, four, good, okay, and finger gun. The subjects repeated a set of a 5-s rest gesture and a 5-s non-rest gesture five times in each round.

The subjects were asked to perform the gestures in the order shown in Figure 4. The recording of a 5-s rest gesture and a 5-s non-rest gesture is referred to as a set. Five repeated sets formed a round. The subjects conducted four rounds sequentially for each non-rest gesture. Between rounds, a 10 s rest interval was also given. After completing four rounds of a given gesture, participants took a 5 min rest for relaxing their muscles before performing a new gesture.

Signals measured from three EMG sensors were recorded simultaneously using LabVIEW. EMG signals have frequencies mostly in the range of 20–500 Hz. Noise in the signals were reduced using two digital filters implemented in the LabVIEW: a bandpass filter (Butterworth, 4th order, 20–500 Hz) and a bandstop filter (Butterworth, 7th order, 59.5–60.5 Hz).

### 2.4. Data Preprocessing

**Figure 5:**
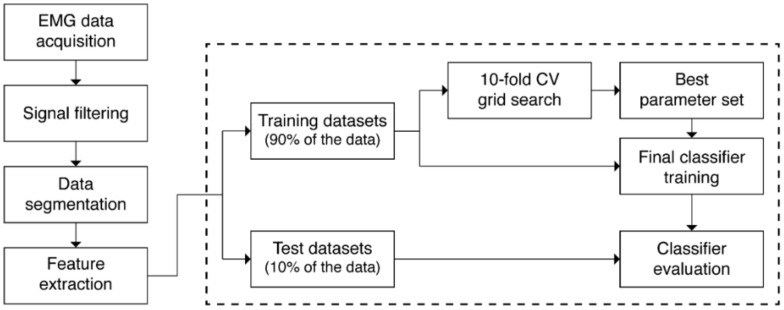
Flow chart of data processing steps.

**Figure 6:**
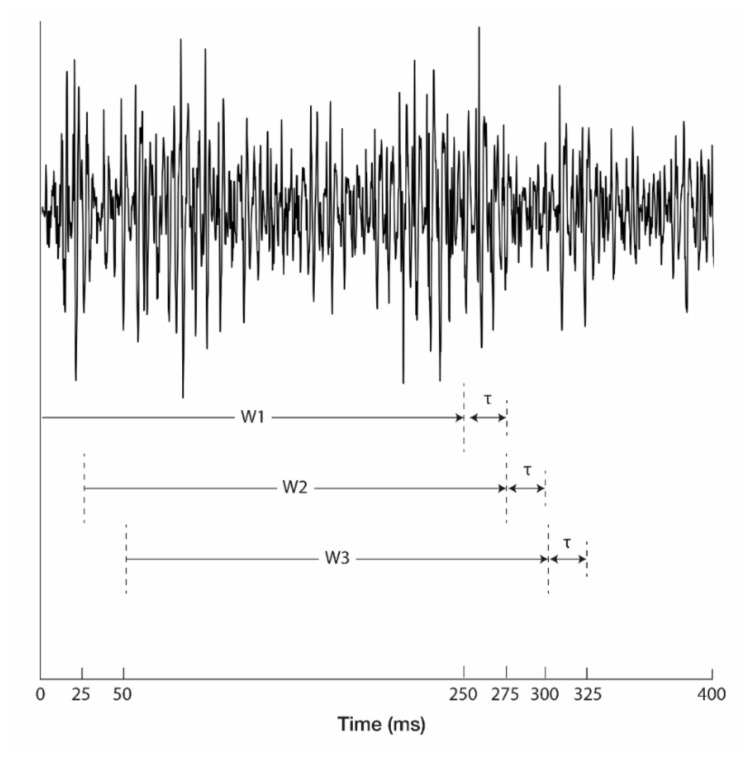
Moving window for EMG signal segmentation on one channel, where the 90% overlapping window technique was applied. W1, W2, and W3 denote the moving windows, which have a length of 250 ms, and *τ* denotes the interval between the windows, which is 25 ms.

Figure 5 shows a flow chart of the data processing steps. An overlapping sliding window was adopted for data segmentation (Figure 6). The length of a moving window was 250 ms, and the window was increased in increments of 25 ms (90% overlapping). Each segmented window was annotated with one of the ten gestures. In particular, to annotate windows in transient states changing from rest to non-rest gestures, a threshold-identifying gesture activation from the rest state was calculated with the first 4-s long-rest data in every round; the highest value was selected as an activation threshold as shown below:

(1) $$\mathrm{Threshold}=\lambda \times {\mathrm{Baseline}}_{max}$$

where λ is an empirical coefficient [37]. The range of λ was determined by evaluating Baseline*_max_* values for each gesture in every round. The smallest Baseline*_max_* among these values was defined as λ = 1. The ratio of the largest to the smallest Baseline*_max_* was defined as the highest value of λ. To find an optimal threshold, we increased λ from 1 to the highest value. Then, the time at which an EMG signal exceeded the threshold was defined as an activation point. Because the three muscles did not activate simultaneously when performing a gesture, the activation points from each channel were different. Therefore, we used the earliest activation time from the three channels. EMG signals were assumed to be in the activated state for 5 s from the activation point. A segmented window was annotated with a non-rest gesture if more than 50% of the window was in an activated state.

### 2.5. Feature Extraction

A total of six TD features, including Hudgins’ features, were extracted from the 250-ms-long segmented datasets: the root mean square (RMS), variance (VAR), mean absolute value (MAV), slope sign change (SSC), zero crossing (ZC), and waveform length (WL) (Table 1) [8,38,39]. These features have been most widely used for real-time EMG signal analyses owing to their relatively low computational requirements. The extracted features were normalized using a standardization method for the classification to achieve a mean of zero and unit variance.

### 2.6. Modeling

We developed personalized classifiers with the datasets obtained from each subject (Appendix A). In each subject dataset, the number of segmented rest gesture observations was nine times higher than the number of non-rest gesture observations, resulting in a class imbalance. When a model is developed from imbalanced data, it cannot perform well on a minority class because training algorithms are designed to reduce errors from inaccurate prediction. If the dataset is highly imbalanced, the algorithm will reduce the error by predicting the majority class and failing to learn the minority class. Most machine learning algorithms perform best when each class has an equal number of samples. Under-sampling is one of the methods used to overcome the data imbalance problem, which matches the number of samples of each class by randomly removing samples from the majority class, as previously applied in an EMG study [40]. Therefore, we under-sampled the rest gesture to obtain a similar number of observations for each class to maximize the performance of the classifiers. We split each subject dataset into a training dataset (90% of the data) and a test dataset (10% of the data, Figure 5).

The following four machine learning methods were used to develop the classifiers and identify the ten gestures in each subject dataset: ANN, SVM, RF, and LR. One of the aims of this study was to test whether traditional TD features can be used for the ANN to develop a multi-class classification model in an EMG-based hand/finger gesture recognition system. Since the pre-calculated TD features were used as input data for classifiers, we chose to adopt a multilayer perceptron model for the ANN.

The parameters for each machine learning model were tuned using stratified ten-fold cross-validation (CV) grid search processes in the training dataset (Figure 5). In brief, the training dataset was randomly divided into ten subparts of equal size. Nine subparts were used for training the classifier with a grid of parameters, and the remaining subpart was used for validation and accuracy evaluation. This process was repeated ten times, with each of the ten subparts used exactly once for validation. Then, ten results from the folds were averaged, and the averaged accuracy values were compared. Because the aim of this study was to build a personalized model for EMG recognition, we focused on evaluating individual performance of the model of each subject. Therefore, we applied a ten-fold CV to each subject model instead of a validation method based on the entire dataset, such as the leave-one-subject-out method.

The following parameters were optimized: the number of hidden layers (2, 3, and 4), the neurons in each layer (300, 600, and 1000), the dropout rate (0.2 and 0.3), and the use of batch normalization for the ANN; kernel (linear and rbf), C (1, 10, 100, and 1000), and gamma (1, 0.1, 0.01, 0.001, and 0.0001) for the SVM; the number of trees (100, 500, 1000) and class weight (balanced subsample and none) for RF; penalty (L1, L2, elasticnet, and none), C (1, 0.1, 0.01, 0.001, and 0.0001), class weight (balanced and none), and solver (lbfgs and saga) for LR. The Adam optimizer, a batch size of 1024, a learning rate of 0.001, and 2000 epochs were used when training and optimizing the ANN classifiers. The optimal parameter, i.e., those that resulted in the highest average accuracy from the ten folds, were selected for each classifier. The final optimized model was built by training the entire training set with the best parameters and was applied to the test dataset (Figure 5).

In addition, we estimated the performance of ANN-based classifiers in real-time decoding. To build a classifier model, data from the first to third round were used as the training dataset and those from the fourth round were used as the test dataset to evaluate the accuracy for each subject.

### 2.7. Statistical Analysis

The classification accuracy for each subject and each machine learning method was evaluated separately. Then, to compare the performance among different machine learning methods, the mean and standard deviation (SD) of the accuracies of all the subjects were used. We evaluated the accuracy of the machine learning method using the one-way analysis of variance (ANOVA) with the Games–Howell post-hoc tests. The variance of the accuracies of the different machine learning methods was evaluated using F-tests with the false discovery rate (FDR) correction. Additionally, we compared the accuracies of the ANN-based classifiers of different feature combinations using the one-way ANOVA with the Games–Howell post-hoc tests. The *P*-values were calculated between groups in the analysis, and *p* < 0.05 was considered statistically significant.

## 3. Results

### 3.1. Classifier Assessment

**Figure 7:**
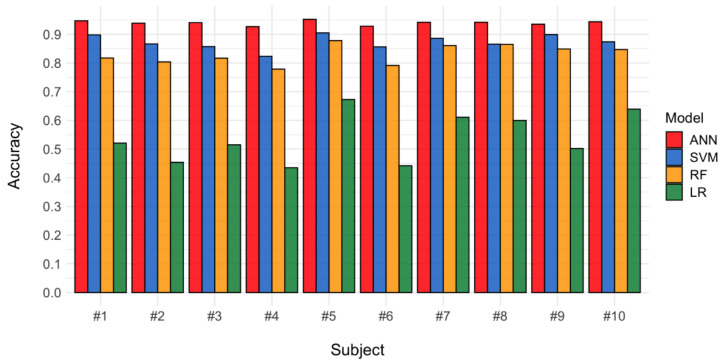
Classifier accuracies for all ten subjects according to the machine learning method.

Ten healthy male subjects participated in the experiment. We developed machine learning-based classifiers to identify the ten gestures for each subject using ANN, SVM, RF, and LR. The optimized classifiers were built with the training datasets based on the best parameters, which were determined using a grid search process, and their performances were estimated using the test datasets (Figure 5). Table 2 shows the best parameters selected by a grid search process for each subject and machine learning method. Table 3 and Figure 7 describe the classification accuracies of the machine learning methods. The mean accuracies (95% confidence intervals) for the ANN, SVM, RF, and LR were 0.940 (0.935–0.945), 0.874 (0.858–0.890), 0.831 (0.809–0.853), and 0.539 (0.483–0.595), respectively. ANN showed the highest accuracy in all subjects. The highest accuracy of 0.952 was obtained in subject #5 with ANN, and the lowest accuracy of 0.435 was obtained in subject #4 with LR.

### 3.2. Statistical Comparison of the Performances of the Machine Learning Methods

**Figure 8:**
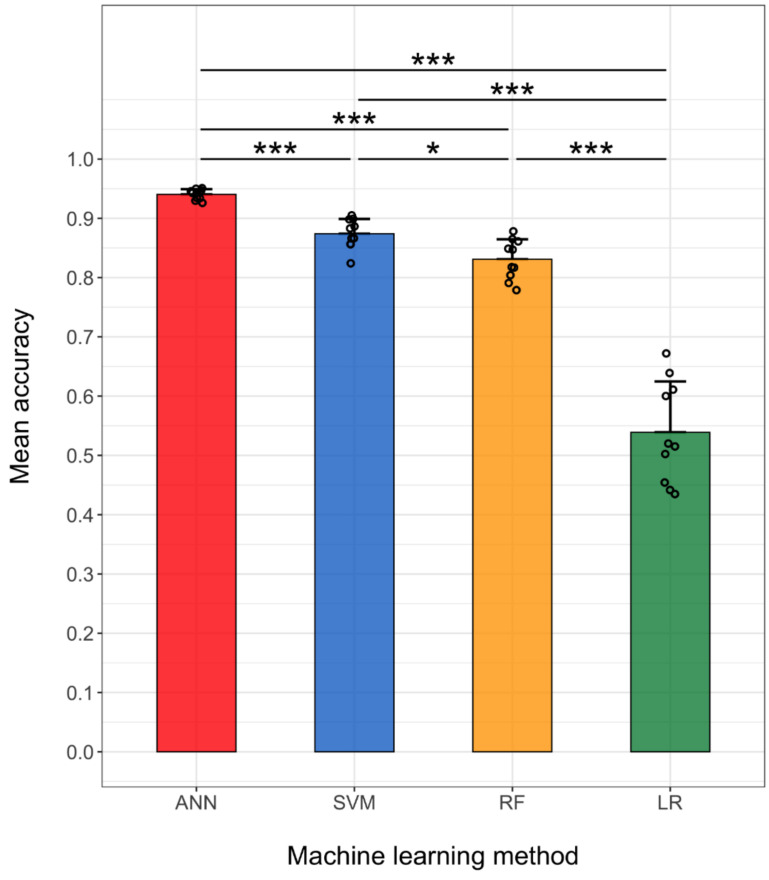
Mean and standard deviation (error bars) of accuracy values obtained with the ANN, SVM, RF, and LR, and plot of the accuracy obtained with each subject (black circles). The machine learning methods significantly affected the accuracy (*p* < 0.001, one-way ANOVA). Post-hoc comparisons between machine learning methods were conducted using the Games–Howell method (* *p* < 0.05, *** *p* < 0.001). The variances in accuracy of the different machine learning methods were determined using F-tests with the FDR correction.

Figure 8 shows the mean and standard deviation of the accuracy of the different machine learning methods obtained with each subject. The one-way ANOVA was conducted to determine whether the main effect of the machine learning method on the accuracy was statistically significant. Because the Levene’s test indicated that the assumption of equal variances was violated (*p* < 0.001), the Welch F-ratio was reported. There was a significant effect of the machine learning method on the accuracy: F (3, 16.367) = 110.23, *p* < 0.001. In addition, Games–Howell post-hoc tests revealed that the ANN achieved significantly higher accuracy than the other three methods (*p* < 0.001 for all three comparisons) and that the SVM achieved significantly higher accuracy than RF (*p* = 0.025) and LR (*p* < 0.001). Furthermore, RF showed significantly higher accuracy than LR (*p* < 0.001).

The distribution of the accuracies (Figure 8) suggests that the ANN and LR had the smallest and largest inter-subject variance in accuracy, respectively, among the methods considered. We used F-tests to statistically compare the variances in accuracy between the methods with *P*-values corrected by the FDR method. Pairwise comparisons of the variances revealed that the ANN had significantly smaller variance than the other three methods (*p* = 0.003 with SVM, *p* < 0.001 with RF and LR), the SVM had significantly smaller variance than LR (*p* = 0.002), and RF showed significantly smaller variance than LR (*p* = 0.012) (Table 4).

### 3.3. Confusion Matrices of ANN-Based Classifiers

**Figure 9:**
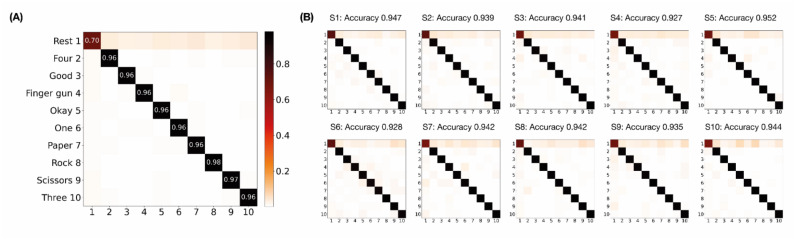
Confusion matrices of ANN-based classifiers in the test datasets. True and predicted labels are shown on the horizontal and vertical axes, respectively. (**A**) Average of all subjects. (**B**) Individual subjects.

The ANN-based classifiers achieved the highest performance, with a mean accuracy of 0.940 in the test datasets (Table 3). Figure 9 presents confusion matrices of the ANN-based classifiers in the test datasets. A combined confusion matrix (Figure 9A) was calculated by averaging entries from confusion matrices of individual subjects (Figure 9B). On average, non-rest gestures were classified with a sensitivity of at least 0.96, while the rest state was classified with a 0.70 sensitivity (Figure 9A). All individual confusion matrices also showed relatively low sensitivity of the rest state compared to the non-rest gestures (Figure 9B). These results suggest that misclassification was mostly due to the prediction of the rest signals as non-rest gestures.

### 3.4. Performance Comparison of ANN-Based Classifiers According to Feature Combinations

**Figure 10:**
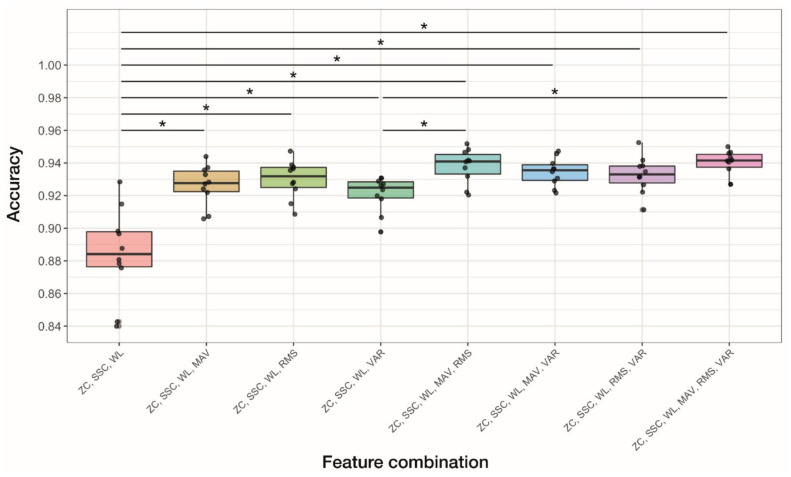
Box plots comparing accuracy of ANN-based classifiers according to feature combinations. The plot shows the median (thick line in the box), interquartile range (the box), range (whiskers), and accuracies obtained from each subject (black dots). The effect of the feature combination on the accuracy was significant (*p* < 0.001, one-way ANOVA). Lines above the box plots display post-hoc comparisons between feature combinations conducted using the Games–Howell method (* *p* < 0.05).

We evaluated the performance of ANN-based classifiers based on different feature combinations. Three features, RMS, VAR, and MAV, are closely related to each other, and redundancy among them has been suggested [7]. To explore the redundancy of these related features, we evaluated the performance of ANN-based classifiers according to the different feature combinations. We used ZC, SSC, and WL as the base feature set and added one, two, or three features selected from the group of RMS, VAR, and MAV. Hence, a total of eight combinations were tested, as shown in Figure 10 and Table 5. The base feature set (ZC/SSC/WL) showed the lowest accuracy, and combining all six features achieved the highest accuracy. One-way ANOVA revealed that the effect of the feature combination on the accuracy was significant (*p* < 0.001). Post-hoc comparisons were run and indicated interesting results (post-hoc graphs in Figure 10). The accuracy was significantly improved by adding features to the base feature set regardless of the combination of RMS, VAR, and MAV. However, after adding one or more features to the base feature set, there was no significant difference in accuracy between any pairs of comparisons except for two cases. The accuracy evaluated from ZC/SSC/WL + VAR was significantly lower than that from ZC/SSC/WL + MAV + RMS and ZC/SSC/WL + MAV + RMS + VAR.

### 3.5. Estimation of Real-Time Performance Using ANN-Based Classifiers

**Figure 11:**
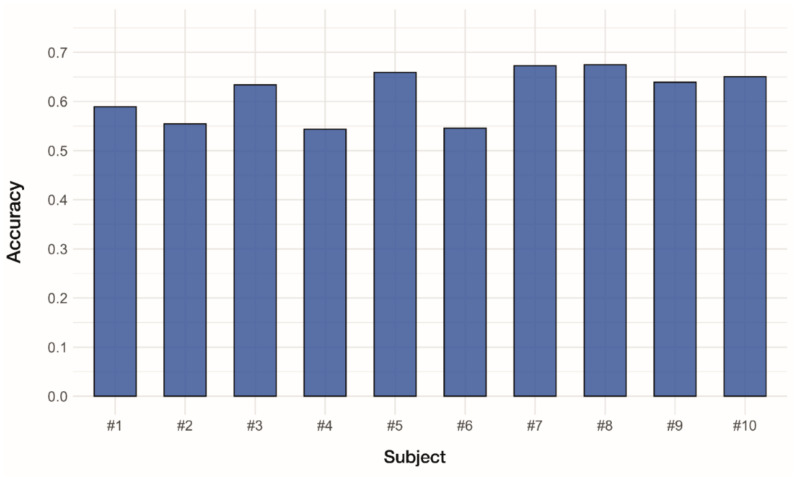
Estimation of real-time performance using ANN-based classifiers.

To estimate the real-time performance of ANN-based classifiers, a classifier model was built with data from the first to the third round, and data from the fourth round were used as the test dataset to evaluate the accuracy. Figure 11 shows the classification accuracy for each subject. The mean accuracy (SD) was 0.616 (0.0530). The highest accuracy of 0.675 was obtained in subject #8, and the lowest accuracy of 0.544 was obtained in subject #4.

## 4. Discussion

We demonstrated the performance of personalized hand/finger gesture classifiers based on TD features only. Four machine learning methods—ANN, SVM, RF, and LR—were implemented to classify ten gestures, including seven IF gestures. The ANN method achieved the highest mean accuracy of 0.940 (Figure 8), suggesting that a relatively large number of gestures can be detected using only three EMG channels. In addition, the ANN-based classifiers showed the lowest variance in the accuracy (Table 4), suggesting that their performances were affected by inter-subject variability in EMG signals significantly less than those of the other methods.

Table 6 shows previous studies in which EMG signals were used to classify hand/finger gestures using machine learning methods. Direct comparisons of the results of different studies are difficult, owing to methodological reasons. Thus, we included recently published, personalized EMG recognition studies that used TD features and focused on IF gestures, and we excluded studies that used commercially available multi-channel wearable devices, which cannot specify the electrode positions relative to the muscles. Table 6 also lists important parameters used: the number of subjects, feature types, the number of features, the number of gestures (N_G_), the number of channels (N_Ch_), the ratio of the number of gestures to that of channels (N_Ch_/N_G_), the window length, machine learning methods used, and accuracy [1].

We chose to use TD features to reduce the time delay due to the computational load, thereby building a more suitable system for real-time detection. TD features are rapid and straightforward to calculate; they can be extracted directly from raw EMG signals without any transformation [41]. Previous studies suggested that TD features performed better in classifying EMG signals in both transient and steady states than the features from other domains [8,9,10]. However, other studies also demonstrated that the combination of features from multiple domains, including FD and TFD, can improve the performance [42]. Our results suggest that TD features can indeed achieve high performance if an algorithm suitable for classification is chosen.

Despite recent advances in ANN technology, to the best of our knowledge, ANN algorithms have been rarely applied to TD features for classifying hand/finger gestures. Classical machine learning methods, such as SVM and KNN, have been applied to TD features and achieved 0.94–0.98 accuracy in classifying four to six gestures [12,14]. Fu et al. used a probabilistic neural network with the features from a TD auto-regressive model to classify eight gestures and achieved a 0.922 accuracy [13]. Qi et al. [16] achieved an accuracy of 0.951 in classifying nine gestures using an ANN algorithm with TD and FD features. However, these studies were performed with a much greater number of channels and features than the present study.

The number of gestures classified in this study was greater than that in most previous studies (Table 6). More importantly, seven out of ten gestures used in this study were associated with the movement of IF (Table 6, Figure 3). Fajardo et al. [18] classified ten gestures, including one IF gesture, but achieved an accuracy of 0.657. Qi et al. [16] classified nine gestures, including four IF gestures, with an accuracy of 0.951. Fu et al. [13] and Sharma and Gupta [15] classified eight IF gestures, but they achieved accuracies lower than that obtained in this study. The ANN classifiers used in this study achieved a high accuracy in the classification of seven IF gestures. Therefore, we achieved high performance in the classification of various IF gestures, which are more challenging to classify than whole-hand and wrist gestures [1], using TD features and ANN algorithms.

We only used three channels to measure EMG signals, and therefore, the ratio of the number of gestures to channels was 3.34, which was higher than that used in most previous studies. Palkowski and Redlarski [12] and Shi et al. [14] used two EMG channels and classified a smaller number of wrist and whole-hand gestures. Sharma and Gupta [15] used three channels to classify nine gestures, but their accuracy was relatively low. Fajardo et al. [18] used a higher ratio of the number of gestures to channels. They used a single channel and classified four to ten gestures. However, in their study, the accuracy decreased significantly as the number of gestures increased; it decreased from 0.952 to 0.657 as the number of gestures increased from four to ten, suggesting that the number of differentiable classes is small if only one channel is used to measure EMG signals. The use of a large number of channels would increase the cost and complexity of signal acquisition hardware and the computation time for classification, which would affect the usability of the recognition system; moreover, the accuracy would not necessarily improve [43]. Therefore, it is important to find an optimal number of channels for the high-accuracy classification of various hand gestures movements. We successfully demonstrated the classification of a large number of gestures using few electrodes, which is advantageous for finger gesture recognition systems.

The entire data acquisition process proceeded for each subject without repositioning the electrodes until the end. Therefore, the retraining of a classifier model to account for the electrode repositioning was not required. However, if the electrodes were repositioned during the measurement, new training might be needed for each repositioning to achieve the best performance in classification. Before attaching the electrodes to each target muscle, we carefully examined the forearm of the subject to find the correct positions to the best of our ability. This effort helped to reduce the effects of electrode location or individual anatomy but did not eliminate them completely. More studies are required in the future to test the effect of electrode repositioning and anatomical variability on the performance of classifiers and to develop a retraining process for adjustment.

Although the ANN showed the highest accuracy in this study, comparisons between algorithms should be bounded by the current dataset and analyzed with caution. The success of a certain algorithm is not solely decided by the superiority of the algorithm itself but is significantly affected by the characteristics of the dataset as well. For example, previous studies demonstrated that ANN models showed lower performance than other classical methods, such as SVM [44]. Therefore, the ANN method proposed in this study may not work well for other EMG datasets. Indeed, a vanilla ANN architecture is prone to overfitting. However, recent ANN techniques, such as dropout and batch normalization, have been employed in EMG studies to overcome overfitting [45]. We also applied these recent techniques to the ANN, which may play an important role in improving the overall performance of the ANN-based classifiers. In the future, we will test additional kernel functions or parameters for SVM models and other machine learning methods for more in-depth comparisons among algorithms.

It is important to note that classifiers based on ANN algorithms showed a significantly lower variance in the accuracy than those based on the other algorithms (Table 4), indicating that their performance was less affected by individual variability. Classifiers for hand/finger gesture recognition should be trained and built based on personalized datasets owing to inter-subject variability in EMG signals [46,47,48,49]. In particular, inter-subject variability between amputees is higher than that between non-amputees. Therefore, achieving consistently high accuracy in subjects with various physiological conditions is a prerequisite for the development of prosthetic control systems based on EMG signals.

The sensitivity for the rest gestures was lower than those for the non-rest gestures in the ANN-based classifier (Figure 9). The confusion matrices indicated that misclassification was caused mainly by the prediction of rest gestures as non-rest gestures. The rest and non-rest gestures were alternated every 5 s during the experiment. Therefore, all transient states in the measured signals were representing transitions between the rest and non-rest gestures. The prediction errors were associated with the EMG signals in these transient states. A similar issue, i.e., prediction errors clustering around a transition zone, was reported in previous studies [50,51,52] and remains challenging. To address this issue, post-processing approaches, such as majority voting and confidence-based rejection, have been suggested and performed, resulting in a decrease in the error rate [53,54]. However, implementing post-processing would increase the overall computation time, forcing trade-off decisions between accuracy and delay.

We compared the accuracy of ANN-based classifiers according to feature combinations (Figure 10). Adding one feature to the base feature set significantly improved the accuracy, suggesting that critical information not present in the base features was provided by RMS, VAR, and MAV. However, adding more than one feature did not lead to a further increase in the accuracy, except for the case of ZC/SSC/WL + VAR. Adding three features resulted in the highest mean accuracy, but statistical significance was observed only when compared to the base feature set and ZC/SSC/WL + VAR. These results suggest that applying RMS, VAR, and MAV simultaneously may cause redundancy in input data for classification [7]. Therefore, although combining all TD features showed the best performance in the current study, redundancy in the input data may need to be addressed, using feature selection methods [55,56]. This issue would have greater significance when the reduction in computational cost is considered crucial in system development.

We estimated the real-time performance of ANN-based classifiers (Figure 11). Compared to the offline decoding (Figure 7), the real-time classification showed a considerable decrease in accuracy with mean accuracy decreased from 0.940 to 0.616. A significant difference between offline and real-time performance has been reported in previous studies. Ortiz-Catalan et al. [57] demonstrated the offline and real-time classification for ten hand gestures using four EMG channels. Using an MLP model, accuracies of 0.912 and 0.609 were achieved for offline and real-time tests, respectively. Similarly, Abbaspour et al. [58] classified ten hand gestures using four EMG channels and demonstrated significant difference in accuracy between the offline and real-time decoding. They tested nine different machine learning algorithms, including MLP, and all of them resulted in a substantial decrease in accuracy. Their MLP model achieved accuracies of 0.917 and 0.698 for offline and real-time tests, respectively. These results suggest that offline performance does not necessarily translate to real-time systems. Abbaspour et al. [58] suggested that the difference in accuracy could be decreased by having subjects practice the gestures. More studies on various aspects, including consistency in muscle contraction, algorithm optimization, and evaluation, will be conducted in the future to improve real-time performance.

It is crucial to find an optimal window length while keeping the data processing time as short as possible. Adopting a longer segmented window would increase the accuracy of the classifiers as more information would be used for gesture recognition, but it would also increase the controller delay and computational burden [53,59]. Previous studies suggested that the window length for EMG signal classification should be less than 300 ms for real-time response and that the optimal length range is 150–250 ms [60,61]. However, few studies reported the window lengths used, so the comparison is difficult (Table 6). With the same window length, Shi et al. [14] achieved a similar accuracy as that achieved in this study, but they only classified four gestures using two EMG channels. Fu et al. [13] and Sharma and Gupta [15] used a 125-ms window but used multiple-domain features and a larger number of features, which may increase the data processing time. Fajardo et al. [18] used a 750-ms window and extracted a large number of features from various domains, which may not be appropriate for real-time applications. In this study, we used a 250-ms window and only extracted TD features to minimize the response time without deteriorating the performance of the classifiers. In the future, we will use a shorter window length to further reduce the response time while considering trade-off between the response time and accuracy.

This study had some limitations. First, only healthy male subjects were recruited. Because we aim to develop personalized classifiers for gesture recognition, more heterogeneous conditions need to be tested, such as females and amputees subjects, or subjects with weak muscles. Particularly, to develop an application for amputees, we may need to implement more EMG channels and more sophisticated models. A previous study demonstrated that different approaches were required for healthy subjects and amputees to optimize classification accuracy [62]. Additionally, because each amputee has different muscle conditions and mobility, the proposed method in this study may not be applicable for practical prosthetic solutions [63]. Second, we did not optimize electrode positions. Our results suggest that a relatively large number of gestures can be detected using only three EMG channels. As the number of channels decreases, the spatial coverage of EMG signals becomes limited, in which case optimizing electrode positions might be critical for improving gesture recognition [64]. A previous study based on fixed electrode placement used two EMG channels to recognize hand/wrist gestures and demonstrated that electrode position optimization improved the classification performance [64]. Because our study was also based on just three channels, optimizing electrode positions might benefit the performance. However, we used fixed electrode positions throughout the experiments and did not test other positions. In addition, the number of subjects should be increased to demonstrate the consistency of the high performance of the classifiers, i.e., low variance in the accuracy, with a heterogeneous population. Finally, the size of and distance between the electrodes (inter-electrode distance, IED) used in this study were larger than recommended [65,66]. The gel area of 201 mm^2^ corresponds to a diameter of 16 mm. Due to the filtering effect of surface electrodes, smaller electrodes with a diameter of 3–5 mm have been recommended. While the IED in this study was 29.4 mm, a shorter IED between 8 to 10 mm has been recommended to reduce crosstalk contamination in EMG signals. To determine the optimum IED, the length of targeted muscle may need to be considered. Therefore, the quality of EMG signals might be considerably affected by the filtering effect and crosstalk. If we use the standard electrode size and IED to measure signals, we may observe different results, which will be further investigated in future studies.

## 5. Conclusions

We developed EMG-based hand/finger gesture classifiers based on ANN, SVM, RF, and LR algorithms, and we tested the classifiers on ten healthy subjects performing ten hand/finger gestures, including seven IF gestures. We achieved a mean accuracy of 0.940 in the classification of gestures with an ANN-based classifier. We only used TD features but achieved a higher ratio of the number of gestures to channels than other similar studies, demonstrating that the method can improve the recognition system usability while reducing the computational burden. The ANN-based classifiers also showed the lowest inter-subject variance in accuracy, suggesting that this method was the least affected by individual variability. In future studies, we will perform additional tests with a larger, more heterogeneous population to further evaluate the performance of the proposed method.

## Figures and Tables

**Table 1:** Equations of the time-domain features used in this study.

Time-Domain Features	Formula
Root mean square (RMS)	$RMS=\sqrt{\frac{1}{N}\overset{N}{\underset{i}{\sum }}x_{i}^{2}}$
Variance (VAR)	$VAR=\frac{1}{N}\sum_{i=1}^{N}{\left(x_{i}-\mu \right)}^{2}$ $,\;\mu =\frac{1}{N}\sum_{i=1}^{N}x_{i}=0$ $VAR=RMS^{2}$
Mean absolute value (MAV)	$MAV=\frac{1}{N}\overset{N}{\underset{i=1}{\sum }}\left|x_{i}\right|$
Slop sign change (SSC)	$\mathrm{SSC}=\sum_{i=2}^{N-1}\left[f\left[\left(x_{i}-x_{i-1}\right)\times \left(x_{i}-x_{i+1}\right)\right]\right]$ $f\left(x\right)=\left\{\begin{array}{c} 1,\;\;\\ 0, \end{array}\;\right.$ $\begin{array}{c} if\;x\;\geq \;0\\ otherwise \end{array}$
Zero crossing (ZC)	$ZC=\overset{N-1}{\underset{i=1}{\sum }}\left[sgn\left(x_{i}\times x_{i+1}\right)\cap \left|x_{i}-x_{i+1}\right|\geq threshold\right]$ $sgn\left(x\right)=\left\{\begin{array}{c} 1,\;\\ 0,\; \end{array}\right.$ $\begin{array}{c} if\;x\;\geq \;threshold\\ otherwise \end{array}$
Waveform length (WL)	$WL=\overset{N-1}{\underset{i=1}{\sum }}\left|x_{i+1\;}-x_{i}\right|$

*N*: number of samples used for calculation; $x_{i}$: $i^{th}$ sample of measurement; the windows used for calculation of features are shown in Figure 6 (*N* = 500 with 250 ms window).

**Table 2:** Best parameters selected by a grid search process for each subject and machine learning method.

		Subject									
Method	Parameter	#1	#2	#3	#4	#5	#6	#7	#8	#9	#10
ANN	Number of hidden layers	4	3	4	4	3	4	3	4	4	4
	Number of neurons	1000	1000	1000	1000	1000	1000	1000	1000	1000	1000
	Dropout rate	0.3	0.3	0.3	0.3	0.2	0.3	0.3	0.3	0.3	0.3
	Batch normalization	applied	applied	applied	applied	applied	applied	applied	applied	applied	applied
SVM	C	10	10	100	100	100	100	10	100	100	100
	Gamma	1	1	1	1	1	1	1	1	1	1
	Kernel	rbf	rbf	rbf	rbf	rbf	rbf	rbf	rbf	rbf	rbf
RF	Number of trees	1000	1000	1000	1000	500	1000	500	1000	1000	1000
	Class weight	BAL	BAL	none	BAL	none	BAL	none	none	none	none
LR	Penalty	L2	none	none	none	none	L2	none	none	none	L2
	C	1	1	1	0.1	1	1	0.001	1	1	1
	Class weight	none	BAL	none	none	none	BAL	None	BAL	none	none
	Solver	lbfgs	saga	lbfgs	saga	lbfgs	lbfgs	saga	lbfgs	lbfgs	lbfgs

**Table 3:** Classifier accuracies for each subject and machine learning method.

	#1	#2	#3	#4	#5	#6	#7	#8	#9	#10	Mean	95% CI
ANN	0.947	0.939	0.941	0.927	0.952	0.928	0.942	0.942	0.935	0.944	0.940	0.935–0.945
SVM	0.898	0.866	0.857	0.824	0.905	0.856	0.883	0.886	0.866	0.899	0.874	0.858–0.890
RF	0.818	0.804	0.817	0.779	0.878	0.791	0.861	0.865	0.849	0.847	0.831	0.809–0.853
LR	0.520	0.454	0.515	0.435	0.672	0.442	0.611	0.600	0.502	0.639	0.539	0.483–0.595

ANN: artificial neural network; SVM: support vector machine; RF: random forest; LR: logistic regression; CI: confidence interval.

**Table 4:** *p*-values from statistical comparisons of the variance in accuracy between machine learning methods. F-tests were used with the FDR correction for *p*-values.

	ANN	SVM	RF
SVM	0.003	-	-
RF	<0.001	0.386	-
LR	<0.001	0.002	0.012

ANN: artificial neural network; SVM: support vector machine; RF: random forest; LR: logistic regression.

**Table 5:** Accuracy of ANN-based classifiers according to feature combinations.

Feature Combination	Mean Accuracy ± SD
ZC/SSC/WL	0.884 ± 0.028
ZC/SSC/WL + MAV	0.926 ± 0.012
ZC/SSC/WL + RMS	0.930 ± 0.011
ZC/SSC/WL + VAR	0.921 ± 0.011
ZC/SSC/WL + MAV+ RMS	0.938 ± 0.011
ZC/SSC/WL + MAV + VAR	0.934 ± 0.009
ZC/SSC/WL + RMS + VAR	0.933 ± 0.011
ZC/SSC/WL + MAV + RMS + VAR	0.940 ± 0.008

ZC: zero crossing; SSC: slope sign change; WL: waveform length; MAV: mean absolute value; RMS: root mean square; VAR: variance.

**Table 6:** Recent EMG-based hand/finger gesture recognition studies that used TD features and focused on IF gestures. Studies that used commercially available multi-channel wearable devices were excluded.

Reference	Number of Subjects	Feature Types	Number of Features	Number of Gestures (N_G_)	Number of Channels (N_Ch_)	N_G_/N_Ch_	Window Length	ML Method	Accuracy
Palkowski & Redlarski, 2016 [12]	N/A	TD	6	6 (2 W + 2 WH + 2 IF)	2	3	N/A	SVM	0.981
Fu et al. 2017 [13]	5	TD-AR	65	8 (8 IF)	6	1.33	125 ms	PNN	0.922
Shi et al., 2018 [14]	13	TD	8	4 (WH + 3 IF)	2	2	250 ms	KNN	0.938
Sharma & Gupta, 2018 [15]	4	TD, FD	33	9 (8 IF + R)	3	3	125 ms	SVM	0.901
Qi et al., 2020 [16]	N/A	TD, FD	64	9 (2 WH + 2 W + 4 IF + R)	16	0.56	N/A	ANN	0.951
Arteaga et al., 2020 [17]	20	TD, FD	24	6 (5 IF + WH)	4	1.5	N/A	KNN	0.975
Fajardo et al., 2021 [18]	N/A	TD, FD, TFD, features extracted by CNN	198	10 (6 W + 3 WH + IF, highest *)	1	10	750 ms	CNN	0.657
				4 (lowest *)		4			0.952
This study	10	TD	18	10 (2 WH + 7 IF + R)	3	3.34	250 ms	ANN	0.940

TD: time domain; AR: auto-regressive; FD: frequency domain; TFD: time–frequency domain; W: wrist; WH: whole hand; IF: individual finger; R: rest state; ML: machine learning; SVM: support vector machine; PNN: probabilistic neural network; KNN: k-nearest neighbors; ANN: artificial neural network; CNN: convolutional neural network; N/A: not applicable.* The study carried out by Fajardo et al. [18] tested four to ten gestures, but the results for the highest and the lowest numbers of gestures were listed.

## Data Availability

Not available.

## Appendix A

The codes for modeling are available at https://github.com/Bioelectronics-Laboratory/EMG_hand_finger_gestures_classification (accessed on 29 December 2021).
